# Nivolumab‐induced liver injury with a steroid‐refractory increase in biliary enzymes, in a patient with malignant mesothelioma: An autopsy case report

**DOI:** 10.1002/ccr3.5174

**Published:** 2021-12-23

**Authors:** Kazumori Arai, Masanori Matsuda, Hiromasa Nakayasu, Shiori Meguro, Takafumi Kurokami, Aki Kubota, Tomohiro Iwasaki, Makoto Suzuki, Shinya Kawaguchi, Toshihide Iwashita

**Affiliations:** ^1^ Department of Pathology Shizuoka General Hospital Shizuoka Japan; ^2^ Department of Gastroenterology Shizuoka General Hospital Shizuoka Japan; ^3^ Department of Respiratory Medicine Shizuoka General Hospital Shizuoka Japan; ^4^ Department of Regenerative and Infectious Pathology Hamamatsu University School of Medicine Hamamatsu Japan

**Keywords:** autopsy, endothelialitis, immune checkpoint inhibitors, immune‐related adverse events, liver, necrosis, nivolumab

## Abstract

This is the first autopsy report of hepatotoxicity from nivolumab immunotherapy for malignant mesothelioma. The increase in levels of biliary enzymes and randomly distributed endothelial damage were steroid‐refractory, but second‐line option was abandoned because of cachexia. Further discussions are needed regarding the customized management of immune‐related toxicities.

## Graphical abstract

Further discussions are needed to manage immune‐related hepatotoxicity with a steroid‐refractory increase in biliary enzymes and/or cancer complications. Management tailored to the conditions of individuals will be important in future.

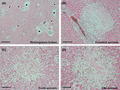

## 1 INTRODUCTION

Nivolumab (NIVO) is a commonly used immune checkpoint inhibitor (ICI) that binds and blocks programmed cell death‐1 (PD‐1), which reinvigorates exhausted autoreactive CD8+ cytotoxic T cells, resulting in immune‐related adverse events (irAEs) in some patients.^1^ The liver has a unique mechanism of immune tolerance, which is essential to avoid excessive immune responses.^2^ Because the PD‐1 pathway is also involved in this mechanism,^2^ the liver is a well‐known target organ of irAEs.^1^, ^3^, ^4^ Recently, we encountered an autopsy case of malignant mesothelioma, with hepatic irAEs induced by NIVO monotherapy. Mesothelioma is an aggressive cancer with a poor outcome, and the treatment options were limited before the introduction of ICI therapy.^5^

To date, the pathological findings of hepatic irAEs, have been based on biopsy tissues.^4^, ^6^, ^7^, ^8^ To our knowledge, there are no autopsy reports detailing the histological changes associated with hepatic irAEs caused by NIVO monotherapy. In the present case, liver enzymes did not normalize throughout the course despite frequent steroid treatments, and biliary enzymes were particularly steroid‐refractory. In addition, as the cancer progressed, the patient's general condition worsened, and the second‐line option was abandoned. Current management guidelines for hepatic irAEs are of high quality, commonly with serum levels of transaminases and/or total bilirubin as the grading indicators. However, they do not include information regarding the management of a steroid‐refractory increase in levels of biliary enzymes and of patients with complications of aggressive cancer.^9^, ^10^, ^11^

## 2 CASE HISTORY

**FIGURE 1:**
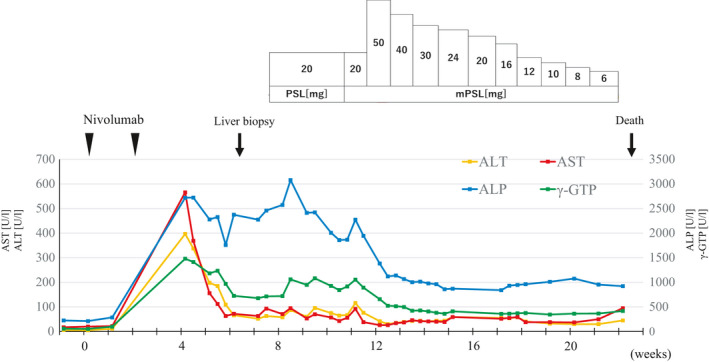
The time courses of assessment of hepatic enzyme levels and steroid treatments. On the fifteenth day after the start of the second course of nivolumab therapy, the liver enzymes were rapidly and markedly elevated. After the discontinuation of nivolumab, transaminases decreased rapidly, but did not normalize throughout the course. Biliary enzymes persisted at high levels throughout the course, despite discontinuation of nivolumab and subsequent steroid treatments. ALP, alkaline phosphatase; ALT, alanine aminotransferase; AST, aspartate aminotransferase; γ‐GTP, γ‐glutamyltranspeptidase; PSL, prednisolone; mPSL, methylprednisolone

A 76‐year‐old man presented with dyspnea persisting for 6 months. He had no family history of cancer. Chest radiograph showed substantial left pleural effusion, and chest computed tomography (CT) demonstrated extensive thickening of the left parietal pleura. A full‐body CT scan did not reveal any metastatic lesions. Parietal pleural biopsy revealed biphasic malignant mesothelioma. The patient received various chemotherapy treatments for 67 months, which included 3 cycles of cisplatin and pemetrexed, 3 cycles of carboplatin and pemetrexed, 27 cycles of pemetrexed alone, and 2 cycles of gemcitabine and 10 cycles of radiation. However, the tumor showed repeated remission and recurrence, resulting in multi‐organ metastases including dissemination of cells to the left lung, bones, parabronchial lymph nodes, and peritoneum. The patient's performance status dropped to 2. The best supportive care was being considered as there was no effective chemotherapy. Around the same time, NIVO monotherapy as a second‐line treatment for patients with malignant mesothelioma^12^ was approved to be covered under insurance. Approximately 5 months after the last chemotherapy, the patient was initiated on NIVO monotherapy (NIVO 240 mg/body intravenously every 2 weeks). However, on the 15th day after the start of the second course of NIVO therapy, a rapid increase in the levels of liver enzymes was noted (alanine transferase [ALT], 397 U/L [normal range, 10–42 U/L]; aspartate aminotransferase [AST], 566 U/L [normal range, 13–30 U/L]; alkaline phosphatase [ALP], 2721 U/L [normal range, 38–113 U/L]; gamma‐glutamyl transpeptidase [γ‐GTP], 1481 U/L [normal range, 13–64 U/L]; Figure 1). The total bilirubin level was 0.6 mg/dL (normal range, 0.4–1.5 mg/dl); furthermore, it was consistently within the normal range at 0.4–0.7 mg/dl throughout the subsequent clinical course. The patient showed no symptoms of hepatitis and was negative for markers for hepatitis A virus, hepatitis B virus, hepatitis C virus, cytomegalovirus (CMV), and Epstein‐Barr virus (EBV). In addition, serum immunoglobulin G (IgG), IgG4, and IgM levels were normal, and anti‐nuclear, anti‐mitochondrial, and anti‐smooth muscle antibodies were all negative. The patient had no history of alcohol consumption. On abdominal CT, no obstructive lesions were detected in the extrahepatic and intrahepatic bile ducts, gallbladder stones were not observed, and no significant changes were noted in the pancreas and duodenal papilla. Bile secretion appeared good on examination of the biliary scintigram. These findings suggested intrahepatic cholestasis. Based on the above clinical data, NIVO‐induced hepatic irAEs were suspected, and the third course of NIVO therapy was abandoned. To verify the diagnosis of hepatic irAEs, a liver biopsy was performed. The biopsy period corresponded to the 47^th^ day from the start of the first course of NIVO therapy. Biopsy revealed various pathological findings, the details of which are described under the section, “Liver pathology.” The severity of hepatic irAEs was graded based on the Common Terminology Criteria for Adverse Events of the National Cancer Institute, version 5.0.^13^ When the liver enzymes increased rapidly, the serum levels of both ALT and AST (transaminases) corresponded to grade 3, and those of both ALP and γ‐GTP (biliary enzymes) corresponded to grade 4.^13^ However, after the discontinuation of NIVO therapy, the serum levels of transaminases rapidly decreased to grade 2 or 1. In contrast, biliary enzyme levels showed a slight decreasing tendency once, but persisted at grade 3 (Figure 1). Based on the treatment for grade 2 irAEs, the patient was started on 20 mg/day oral prednisolone (PSL). The recommended initial dose is 0.5–1 mg/kg/day,^10^, ^11^ at which time the patient weighed approximately 35 kg and was 160 cm in height. Even after continuing the steroid treatment for 3 weeks, the serum levels of transaminases did not improve completely from grade 2 to grade 1 or less. In addition, biliary enzyme levels remained at grade 3 (Figure 1). Therefore, the treatment has been changed to comply with grade 3 irAE management.^10^ Methylprednisolone (mPSL) was initially administered intravenously at 20 mg/day for 5 days and then at 50 mg/day for 7 days (recommended initial dose of 1–2 mg/kg/day^10^, ^11^), and its dose was gradually tapered. The treatment was administered until just before death, and eventually, mPSL was administered for 78 days (total amount 1672 mg). During this treatment, the serum levels of transaminases improved from grade 2 to grade 1, but did not normalize. In addition, biliary enzymes persisted at grades 2 or 3 (Figure 1). At this time, the patient had no signs of infectious diseases, and the second‐line option with mycophenolate mofetil (MMF) was considered.^4^, ^10^, ^11^, ^14^ However, the patient developed cachexia, due to peritoneal dissemination and multi‐organ metastases, and his general condition continued to worsen. Administration of MMF was abandoned due to high risk of infection. Approximately 5.5 months after the start of the first course of NIVO therapy, the patient died of advanced cachexia and autopsied. The patient did not have disseminated intravascular coagulation. At autopsy, the tumor had extensively disseminated to the peritoneum (maximum diameter of 8 cm) and had metastasized to multiple organs (maximum diameter of 6.5 cm), including the left lung and left kidney. However, therapeutic effects were minimal. There were no lesions in any of the organs suggesting a serious infection.

### 2.1 Liver pathology

#### 2.1.1 Biopsy

**FIGURE 2:**
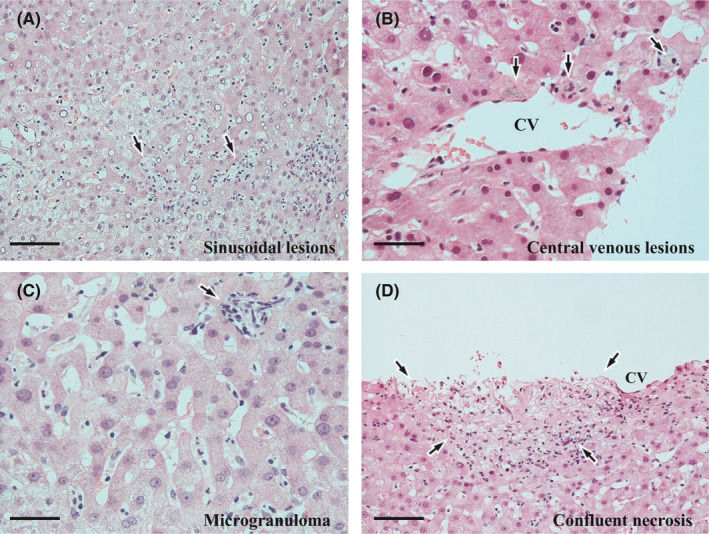
Histopathological findings of the biopsy liver I. (A) Sinusoidal inflammatory infiltrates with spotty necrosis (arrows). Hematoxylin and eosin (H&E) stain. Scale bar: 100 μm. (B) Slight inflammatory infiltrates underneath the endothelium of the central vein (CV), and perivenular ceroid‐laden macrophages (arrows). H&E stain. Scale bar: 50 μm. (C) Sinusoidal infiltration of small lymphocytes and neutrophils, and a microgranuloma (arrow). H&E stain. Scale bar: 50 μm. (D) Eccentric confluent necrosis (surrounded by arrows) adjacent to the central vein (CV). H&E stain. Scale bar: 100 μm

In the lobules, mild, but diffuse, infiltration of sinusoidal inflammatory cells was observed (Figure 2A). Infiltration was also observed underneath the endothelium of some of the central veins (Figure 2B). Inflammatory cells mainly comprised of small lymphocytes and neutrophils (Figure 2C), whereas histiocytes were mostly distributed as small aggregates or microgranulomas (Figure 2C). In addition, scattered spotty necrosis (Figure 2A) and partial sinusoidal dilation were observed. Furthermore, as a notable change, confluent necrosis (CN) was eccentrically found in one of central zones (Figure 2D). These findings indicated acute lobular hepatitis. In addition, infiltration of ceroid‐laden macrophages was also seen adjacent to some of the central veins (Figure 2B), representing an old centrilobular hepatocyte injury. Hepatocellular cholestasis was slight.

**FIGURE 3:**
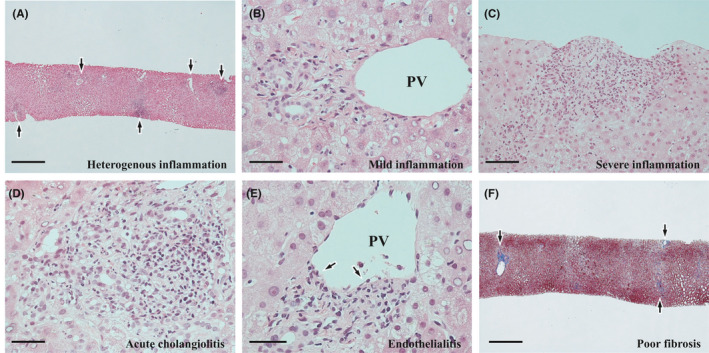
Histopathological findings of the biopsy liver II. (A) Infiltration of inflammatory cells with variable degrees among the portal areas (arrows). Hematoxylin and eosin (H&E) stain. Scale bar: 1 mm. (B) Portal area with mild infiltration of inflammatory cells. PV: portal vein branch. H&E stain. Scale bar: 50 μm. (C) Severe portal inflammation with interface hepatitis. H&E stain. Scale bar: 100 μm. (D) Ductular reaction with neutrophil infiltration. H&E stain. Scale bar: 50 μm. (E) Portal endothelialitis with endothelial detachment (arrows). PV: portal vein branch. Scale bar: 50 μm. (F) Mild or near‐absent fibrosis of the portal areas (arrows). Azan stain. Scale bar: 1 mm

The changes in the portal areas varied among the areas (Figure 3A), and the areas with slight changes were also mixed (Figure 3B). In the active areas, the infiltrating cells were mainly small lymphocytes. Furthermore, the following changes accompanied to varying degrees: interface hepatitis (Figure 3C), ductular reaction with neutrophil infiltration (acute cholangiolitis) (Figure 3D), endothelialitis (Figure 3E). No ductopenia was seen. Portal fibrosis was slight or absent (Figure 3F).

**FIGURE 4:**
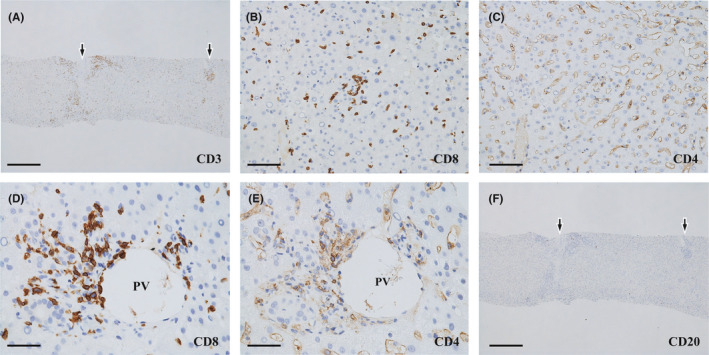
Immunohistochemistry of the biopsy liver. (A) Many positive cells can be seen in both the portal areas (arrows) and lobules. CD3 immunostain. Scale bar: 500 μm. (B) The majority of lymphocytes infiltrating the lobules were positive. CD8 immunostain. Scale bar: 100 μm. (C) Image corresponds to (B). There are only a few positive infiltrating lymphocytes, whereas sinusoidal endothelia and Kupffer cells are positive, rimming the sinusoids. CD4 immunostain. Scale bar: 100 μm. (D) Many portal lymphocytes, including those invading the endothelium, are positive. PV: portal vein branch. CD8 immunostain. Scale bar: 500 μm. (E) Image corresponds to (D). There are fewer positive lymphocytes than CD8+ cells (D). PV: portal vein branch. CD4 immunostain. Scale bar: 50 μm. (F) Image corresponds to (A). There are very few positive cells compared to the CD3+ cells (A). Arrows indicate portal areas. CD20 immunostain. Scale bar: 500 μm

Immunohistochemically, infiltrating lymphocytes consisted mainly of CD3+ and CD8+ T cells in both the lobules and portal areas (Figure 4A‐E). CD8+ T cells were also predominant in portal endothelialitis (Figure 4D and E). CD20+ B cells were rarely observed (Figure 4F). Immunostaining for CMV and human herpes virus and in situ hybridization for EBV early RNA were negative.

#### 2.1.2 Autopsy

##### Gross findings

**FIGURE 5:**
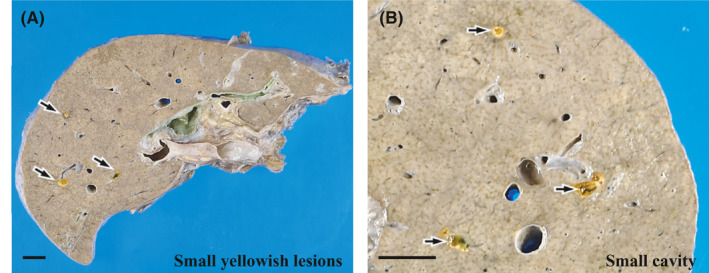
Macroscopic findings in the autopsy liver. (A) No metastases are detected inside the liver, whereas a few random small yellowish lesions (arrows) are visible. Scale bar: 1 cm. (B) Magnified image of (A). Individual lesions (arrows) include a small cavity. Scale bar: 1 cm

The liver was atrophied and weighed 650 g. No metastases were detected; however, a few yellowish lesions, 2–4 mm in diameter, were randomly observed (Figure 5A). Individual lesions involved a small cavity (Figure 5B). Neither stone nor stenosis was found in the segmental bile ducts, hepatic ducts, or in the common bile duct.

##### Microscopic findings

**FIGURE 6:**
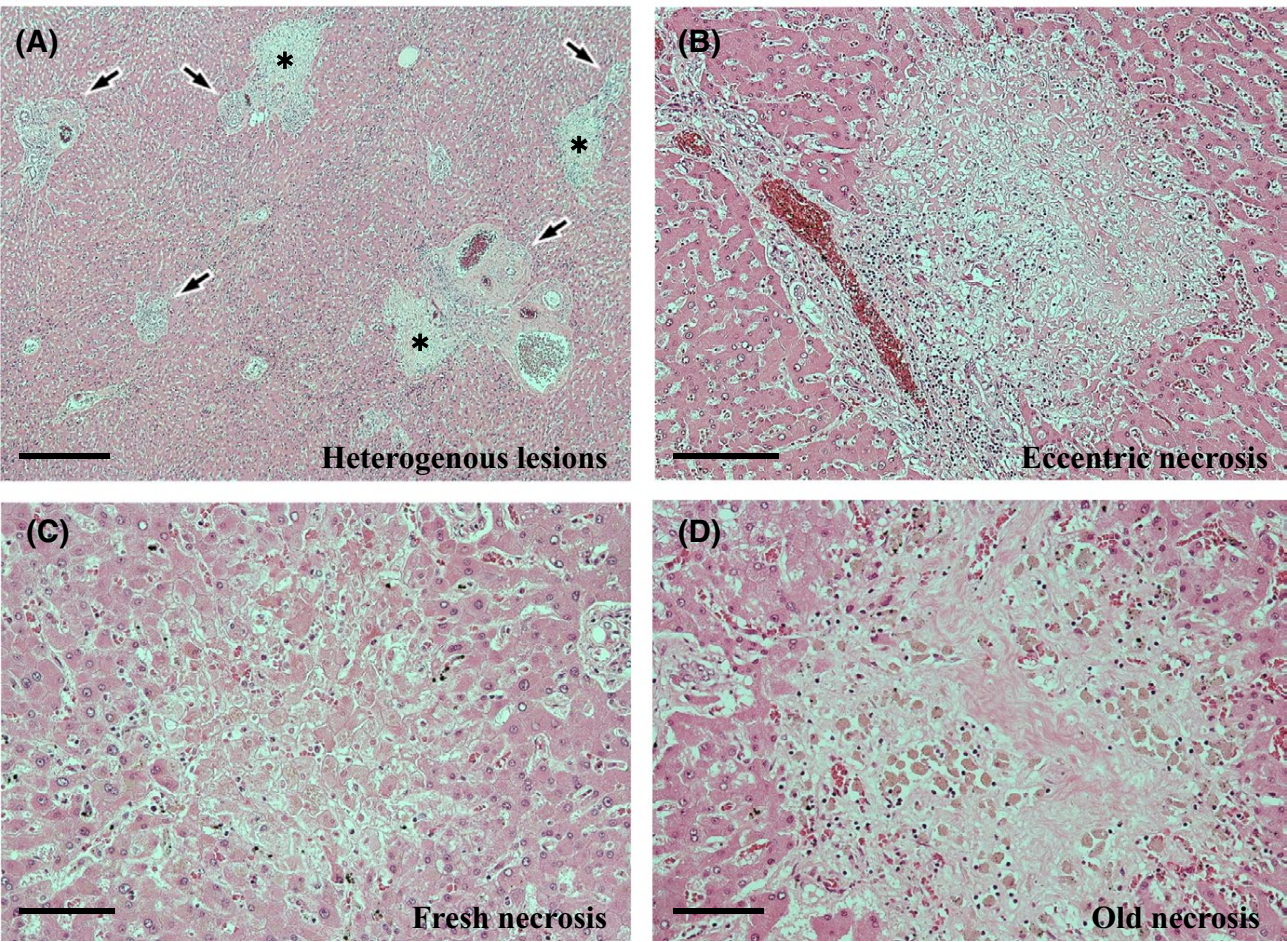
Histopathological findings of the autopsy liver I. (A) Inflammatory reactions are heterogenous among the portal areas (arrows), and periportal confluent necrosis (CN) (asterisks) is randomly scattered. Some lobules lack noticeable findings. Hematoxylin and eosin (H&E) stain. Scale bar: 500 μm. (B) CN is eccentric, with many inflammatory cells infiltrating inside and around it, and inflammatory infiltrates with ductular reaction are seen in the portal area. H&E stain. Scale bar: 200 μm. (C) Coagulative CN with poor inflammatory cell infiltration. H&E stain. Scale bar: 100 μm. (D) CN replaced by fibrosis with many pigmented macrophages H&E stain. Scale bar: 100 μm

**FIGURE 7:**
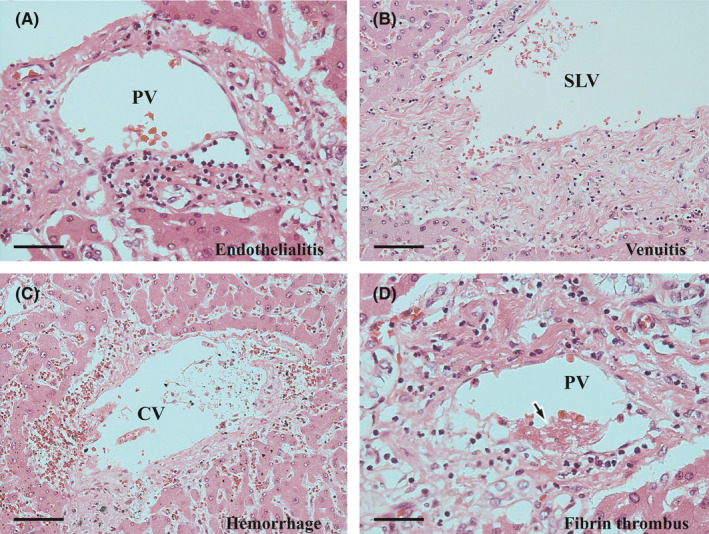
Histopathological findings of the autopsy liver II. (A) Endothelialitis of the portal vein branch (PV), with detachment of the endothelium. Hematoxylin and eosin (H&E) stain. Scale bar: 50 μm. (B) Venuitis of the sublobular vein (SLV). H&E stain. Scale bar: 100 μm. (C) Perivenular hemorrhage. CV: central vein. H&E stain. Scale bar: 100 μm. (D) Fibrin thrombus (arrow) in the portal vein branch (PV) with endothelialitis. H&E stain. Scale bar: 50 μm

**FIGURE 8:**
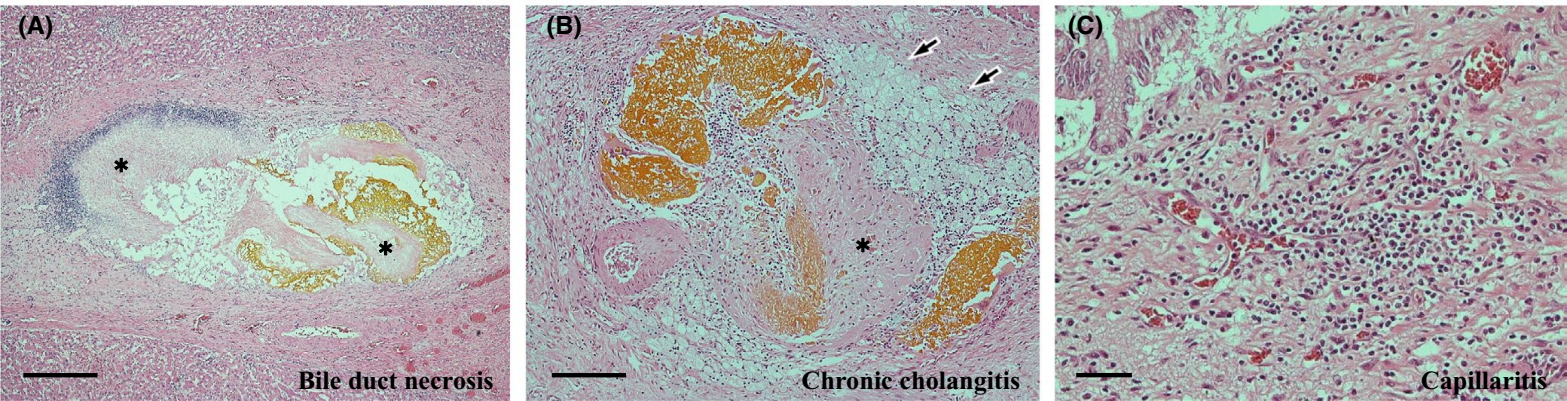
Histopathological findings of the autopsy liver III. (A) Chronic cholangitis of an area of the bile duct with massive necrosis (asterisks) on the luminal side. Hematoxylin and eosin (H&E) stain. Scale bar: 500 μm. (B) Infiltration of lymphocytes and foamy macrophages (arrows) and organization (asterisk) of the bile plug. H&E stain. Scale bar: 200 μm. (C) Infiltration of lymphocytes in the peribiliary capillary plexus. H&E stain. Scale bar: 50 μm

Both sinusoidal inflammatory infiltrates and the activity of interface hepatitis diminished, and the microgranulomas had disappeared. Nevertheless, some other active changes remained. The changes were randomly distributed and the degree of their manifestation was not uniform. The most notable active change was CN. The CN was scattered throughout each of the zones (Figure 6A), although it was slightly predominant in the periportal zone; furthermore, it was eccentrically distributed in the periportal or central zone (Figure 6B). CN is often accompanied by infiltration of various inflammatory cells, both inside and outside (Figure 6B). However, some CN was coagulative, with poor inflammatory cell infiltration (Figure 6C), or was replaced by fibrosis with pigmented macrophages (Figure 6D). The second most noticeable change was the presence of venous lesions. Endothelialitis was predominantly observed in the portal vein branches (Figure 7A). In the hepatic venules, moderate venulitis, with or without endothelialitis, was found in some of the sublobular veins (Figure 7B), whereas perivenular hemorrhage was seen in some of the central veins (Figure 7C). In addition, fibrin thrombi and endothelial detachment were observed in some of the portal vein branches and hepatic venules (Figure 7D). Tumor embolism could not be confirmed. Third, chronic cholangitis with massive necrosis was found in the bile duct area (Figure 8A). The inflammatory foci had a bile plug (Figure 8A and B) and foamy macrophage aggregates (Figure 8B) and were few and localized, corresponding to macroscopic yellowish lesions (Figure 5). Small lymphocyte infiltration was evident in the peribiliary capillary plexus (Figure 8C). No fibrosis was observed in the bile ducts corresponding to the efferent tract.

In the portal areas, inflammatory infiltrates and acute cholangiolitis were also persistent in some of the areas and were often seen in the portal areas with periportal CN (Figure 6B). Slight or no portal fibrosis was seen. The portal changes diminished toward the hepatic hilum. No notable irAEs were noted in organs other than the liver.

Immunohistochemical findings of the infiltrating lymphocytes were similar to those in the biopsy tissue. In the foci of necrotizing cholangitis, the infiltrating lymphocytes were also predominantly CD8+ T cells. The three abovementioned viruses could not be detected either.

## 3 DISCUSSION

In the present case, the patient had received high doses of chemotherapy for a long time due to the aggressive nature of cancer. Tumor necrosis due to chemotherapy or overgrowth beyond the blood supply, may have caused damage‐associated molecular patterns, which also predisposes the liver to inflammation.^15^ In addition, this patient's general condition deteriorated. Consequently, he had various parameters that were prone to inflammation. From these, it is not clear whether all of the hepatic lesions can be considered NIVO‐induced irAEs.^16^ Furthermore, hepatic lesions are difficult to distinguish from nonspecific ones.^3^, ^16^ However, endothelialitis, eccentric CN, and bile duct injury, including acute cholangiolitis, were all randomly scattered in the present case. This heterogeneity of distribution could only be confirmed by autopsy. They have been described in previous reports of hepatic irAE, but each is difficult to distinguish from nonspecific lesions. Such randomly distributed lesions are usually not encountered in the autopsied liver.^17^ Therefore, the heterogeneity of lesion distribution is considered to be the most characteristic pathological finding of NIVO‐induced hepatic irAEs.

Whether the pathological findings presented in this case are common to all patients with NIVO‐induced hepatic irAEs, cannot be mentioned, because there have been no autopsy cases to date. Previous reports using biopsy have suggested that the pathological findings of hepatic irAEs, especially of anti‐PD‐1‐induced irAEs, vary greatly among individuals.^4^, ^7^, ^8^ In the present case, autopsy findings suggested heterogeneity of lesions even within a single organ. In case of hepatic irAEs, biopsy is useful for assessing the severity of the liver damage and efficacy of treatment strategies.^3^, ^4^ However, in practice, the quantity of biopsy tissue is insufficient for examining the distribution of lesions. Even if CN is scattered, it is not always reliably collected by biopsy because of its random distribution. Thus, there is a possibility of lesions not being adequately harvested on the biopsy.

Hepatic irAEs are closely associated with the cytotoxicity of CD8+ T cells.^7^, ^8^ Coagulative CN, with poor inflammatory infiltrates, suggests local ischemia rather than the direct injury to hepatocytes caused by CD8+ T cells.^18^, ^19^ Sinusoids are vascular systems with a slow blood flow.^20^ Fibrin thrombi caused by endothelialitis are considered to become emboli in the sinusoids and create random CN.^19^, ^21^ Less zone‐selective CN in hepatic irAEs has been suggested, previously.^8^ In the present case, no severe fibrosis was found even in the autopsy liver. This also suggests that individual CN occurred transiently. Necrosis of inflamed bile ducts is also thought to have resulted from the damage caused by CD8+ T cells to the feeding vessels.^22^

In the present case, the direct injury to hepatocytes caused by CD8+ T cells, such as lobular hepatitis and interface hepatitis, is presumed to have been diminished by the cessation of NIVO. In contrast, endothelial damage, subsequent ischemic/necrotic lesions and bile duct injury did not respond even to frequent steroid therapies; hence, such lesions are considered steroid‐resistant.

Persistence of increased biliary enzyme levels after the NIVO therapy is considered to be due to prolonged acute cholangiolitis and chronic cholangitis. The rapid increase in transaminases immediately after two courses of NIVO therapy possibly originates from scattered centrilobular hepatocyte injury and CN. However, the subsequent decrease in transaminase levels appears to be a change that does not match the scattered CN, which persists even at autopsy. This discrepancy is apparently due to the total amount of necrotic hepatocytes, but we cannot interpret it properly. For physicians, persistence of scattered CN, without marked elevations of transaminases, interferes with the consideration of liver conditions.

Corticosteroids are not associated with overall survival or shorter time to failure of cancer immunotherapy.^3^, ^23^ It may have been necessary to switch oral PSL treatment to a high‐dose steroid therapy earlier.^10^, ^23^ However, during the oral PSL treatment, transaminase levels did not improve from grade 2 but were not within grade 3.^9^, ^11^ Responses to the treatment for hepatic irAEs vary greatly among individuals.^3^, ^7^, ^24^ Generally, corticosteroids should be used at the lowest dose^23^; furthermore, high‐dose steroid administration does not always have beneficial effects for some patients with irAEs.^23^, ^25^ In the present case, the patient received the best supportive care for chemotherapy‐resistant aggressive cancer, and his performance status was 2. Considering the side effects of steroids such as infections, it was difficult to determine when to switch to a high‐dose steroid treatment.^3^, ^7^, ^23^, ^24^, ^25^, ^26^ Regarding steroid treatment for patients like the one in the present case, a careful assessment of the balance between benefit and risk, may be very difficult. The biological markers predictive of steroid‐resistance, will be coveted.^25^, ^27^

MMF, a steroid‐sparing agent, is recommended as a second‐line option for irAEs when steroids are ineffective.^9^, ^10^, ^11^ Current management guidelines commonly recommend measuring transaminase and/or total bilirubin levels as the grading indicators. Therefore, the assessment of steroid efficacy will naturally depend on those indicators. It may be debatable to define this case as steroid‐resistant hepatic irAEs.^9^, ^10^, ^11^ The problem of this case was that the increase in biliary enzymes that are not mentioned as grading indicators in the guidelines, was rather steroid‐refractory. In addition, the patient developed cachexia, a complication of cancer. It would have been helpful if such cases were also mentioned in the guidelines.^3^, ^28^ Had this been the case, MMF may have been administered for this patient as well.^25^ Patients require highly personalized management.^10^, ^27^ In the future, management tailored to different conditions of individual patients, will be important.^3^, ^10^, ^11^, ^14^, ^23^, ^24^, ^25^, ^26^, ^27^, ^28^, ^29^, ^30^

This report has some limitations, including the examination focusing only on the liver, poor interpretation of the discrepancy between transaminase transitions and persistent scattered CN, abandoning the second‐line option, and the intrinsic limitations of a case report.

In conclusion, this is the first report of NIVO‐induced hepatic irAEs observed in both biopsy and autopsy tissues. The patient had a malignant mesothelioma. Clinically, an increase in biliary enzymes was rather steroid‐refractory. Histological examinations suggested the following two points: 1) the heterogeneity of lesion distribution is the most characteristic finding of NIVO‐induced hepatic irAEs; 2) endothelial damage, followed by ischemic/necrotic lesions, renders NIVO‐induced hepatic irAEs refractory. Further discussions are needed to manage hepatic irAEs with a steroid‐refractory increase in biliary enzymes and/or cancer complications.

## CONFLICT OF INTEREST

The authors declare no conflicts of interest regarding the publication of this article.

## AUTHOR CONTRIBUTIONS

All authors were involved in the preparation of this manuscript. KA analyzed all pathological data and wrote the initial draft of the manuscript. MM, HN, TK and SK contributed to the analysis and interpretation of clinical data. MM contributed to obtaining informed consent for publication as a case report from the patient's family. HN contributed to obtaining consent for autopsy. SM and MS provided administrative, technical, or material support. AK and T Iwasaki contributed to the analysis and interpretation of the immunohistochemical data. SM and T Iwashita interpreted the data and revised the manuscript critically for important intellectual content. All authors read and approved the final version of the manuscript.

## ETHICAL APPROVAL

This case report was conducted according to the Helsinki Declaration and approved by the Ethics Committee of Shizuoka General Hospital.

## CONSENT

Written informed consent was obtained from the patient's family.

## Data Availability

The authors declare that all relevant data are included in this published article and are available in this paper.
